# *catena*-Poly[[dichloridozinc(II)]-μ-1,1′-(hexane-1,6-di­yl)diimidazole-κ^2^ *N* ^3^:*N* ^3′^]

**DOI:** 10.1107/S1600536809037696

**Published:** 2009-09-26

**Authors:** Wen-Li Bao

**Affiliations:** a Criminal Investigation Department, Jilin Public Security Academy, Changchun 130117, People’s Republic of China

## Abstract

In the structure of the polymeric title compound, [ZnCl_2_(C_12_H_18_N_4_)]_*n*_ or [ZnCl_2_(*L*)]_*n*_, where *L* = 1,1′-(hexane-1,6-di­yl)diimidazole, the Zn^II^ centre is coordinated by two N atoms of two different *L* ligands and by two chloride anions in a distorted tetra­hedral geometry. The organic ligand links adjacent metals to form a polymeric chain along the *c* axis. The chains are further connected into layers parallel to the *bc* plane by inter­molecular C—H⋯Cl hydrogen bonds. Two C atoms of the central hexyl chain are disordered over two positions with site-occupancy factors of 0.5.

## Related literature

For general background on coordination polymers, see: Batten & Robson (1998 ▶). For a related structure, see: Yang *et al.* (2008 ▶).
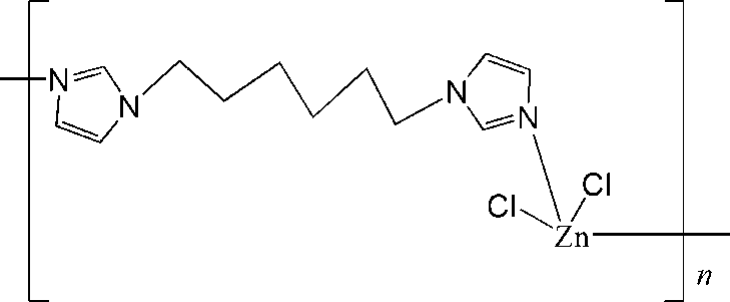


## Experimental

### 

#### Crystal data

[ZnCl_2_(C_12_H_18_N_4_)]
*M* *_r_* = 354.57
Orthorhombic, 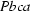

*a* = 11.255 (6) Å
*b* = 11.713 (8) Å
*c* = 23.653 (15) Å
*V* = 3118 (3) Å^3^
*Z* = 8
Cu *K*α radiation
μ = 5.27 mm^−1^
*T* = 293 K
0.31 × 0.25 × 0.22 mm

#### Data collection

Oxford Diffraction Gemini R Ultra diffractometer
Absorption correction: multi-scan (*CrysAlis RED*; Oxford Diffraction, 2006 ▶) *T*_min_ = 0.56, *T*_max_ = 0.85
21446 measured reflections
2778 independent reflections
2080 reflections with *I* > 2σ(*I*)
*R*_int_ = 0.053

#### Refinement

*R*[*F* ^2^ > 2σ(*F* ^2^)] = 0.038
*wR*(*F* ^2^) = 0.106
*S* = 1.03
2778 reflections
190 parameters
2 restraints
H-atom parameters constrained
Δρ_max_ = 0.24 e Å^−3^
Δρ_min_ = −0.39 e Å^−3^

### 

Data collection: *CrysAlis CCD* (Oxford Diffraction, 2006 ▶); cell refinement: *CrysAlis RED* (Oxford Diffraction, 2006 ▶); data reduction: *CrysAlis RED*; program(s) used to solve structure: *SHELXS97* (Sheldrick, 2008 ▶); program(s) used to refine structure: *SHELXL97* (Sheldrick, 2008 ▶); molecular graphics: *SHELXTL* (Sheldrick, 2008 ▶); software used to prepare material for publication: *SHELXTL*.

## Supplementary Material

Crystal structure: contains datablocks global, I. DOI: 10.1107/S1600536809037696/rz2360sup1.cif

Structure factors: contains datablocks I. DOI: 10.1107/S1600536809037696/rz2360Isup2.hkl

Additional supplementary materials:  crystallographic information; 3D view; checkCIF report

## Figures and Tables

**Table 1:** Hydrogen-bond geometry (Å, °)

*D*—H⋯*A*	*D*—H	H⋯*A*	*D*⋯*A*	*D*—H⋯*A*
C3—H3⋯Cl1^i^	0.93	2.77	3.698 (5)	176

Symmetry code: (i) 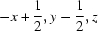.

**Fig. 1.**
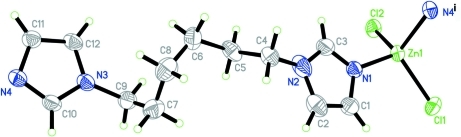
The asymmetric unit of the title compound, showing the atomic numbering scheme and displacement ellipsoids drawn at the 30% probability level. Only one component of the disordered carbon atoms is shown. [Symmetry code: (i) 1/2-x, 1-y, 1/2+z].

**Fig. 2.**
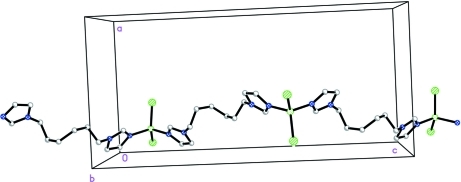
View of the polymeric chain in the title compound. H atoms are omitted for clarity.

## supplementary crystallographic information

### Comment

The design and synthesis of metal-organic coordination polymers are of great interest due to their tremendous potential applications (Batten & Robson, 1998). The selection of the ligands is extremely important because changing their geometries can control the topologies of the resulting coordination frameworks. So far, the rigid rod-like spacer 4,4'-bipyridine is well known in the design of metal-organic polymers. However, flexible N-donor ligands such as 1,1'-(hexane-1,6-diyl)diimidazole (L) have not been so well explored (Yang *et al.*,  2008).

The asymmetric unit of the title compound comprises a zinc(II) cation, a 1,1'-(hexane-1,6-diyl)diimidazole ligand, and two chloride anions (Fig. 1). The metal centre is coordinated by two N atoms of two different *L* ligands, and two Cl anions in a distorted tetrahedral geometry. The C4 and C5 carbon atoms of the hexyl chain are disordered over two positions with site occupancy factors of 0.5. The organic ligand links metal centers at (0.5-x, 1.-y, 0.5+z) and (0.5-x, 1.-y, -0.5+z) to form a polymeric one-dimensional chain (Fig. 2). Adjacent chains are connected by C—H···Cl hydrogen bonds (Table 1) into layers parallel to the  *bc* plane.

### Experimental

Zinc chloride (0.5 mmol) and *L* (0.5 mmol) were placed in water (12 ml), and triethylamine was added until the pH value of the solution was 5.5. The solution was heated in a 23-ml Teflon-lined stainless-steel autoclave at 450 K for 3 days. The autoclave was allowed to cool to room temperature over several hours. Colourless blocks were isolated in about 48% yield.

### Refinement

H atoms were generated geometrically and  refined as riding atoms, with C—H = 0.93-0.96 Å and U_iso_(H) = 1.2 U_eq_(C). Disorder was noted in bridging *L* molecule. Two positions of equal site occupancy factor (0.5) were discerned for the C4 and C5 carbon atoms.

### Crystal data

**Table:**

[ZnCl_2_(C_12_H_18_N_4_)]	*F*(000) = 1456
*M**_r_* = 354.57	*D*_x_ = 1.511 Mg m^−^^3^
Orthorhombic, *P**b**c**a*	Cu *K*α radiation, λ = 1.54184 Å
Hall symbol: -P 2ac 2ab	Cell parameters from 2778 reflections
*a* = 11.255 (6) Å	θ = 3.1–67.2°
*b* = 11.713 (8) Å	µ = 5.27 mm^−^^1^
*c* = 23.653 (15) Å	*T* = 293 K
*V* = 3118 (3) Å^3^	Block, colourless
*Z* = 8	0.31 × 0.25 × 0.22 mm

### Data collection

**Table:**

Oxford Diffraction Gemini R Ultra diffractometer	2778 independent reflections
Radiation source: fine-focus sealed tube	2080 reflections with *I* > 2σ(*I*)
graphite	*R*_int_ = 0.053
Detector resolution: 10.0 pixels mm^-1^	θ_max_ = 67.2°, θ_min_ = 5.4°
ω scans	*h* = −12→13
Absorption correction: multi-scan (*CrysAlis RED*; Oxford Diffraction, 2006)	*k* = −13→12
*T*_min_ = 0.56, *T*_max_ = 0.85	*l* = −28→27
21446 measured reflections	

### Refinement

**Table:**

Refinement on *F*^2^	Primary atom site location: structure-invariant direct methods
Least-squares matrix: full	Secondary atom site location: difference Fourier map
*R*[*F*^2^ > 2σ(*F*^2^)] = 0.038	Hydrogen site location: inferred from neighbouring sites
*wR*(*F*^2^) = 0.106	H-atom parameters constrained
*S* = 1.02	*w* = 1/[σ^2^(*F*_o_^2^) + (0.0617*P*)^2^ + 0.9261*P*] where *P* = (*F*_o_^2^ + 2*F*_c_^2^)/3
2778 reflections	(Δ/σ)_max_ = 0.001
190 parameters	Δρ_max_ = 0.24 e Å^−^^3^
2 restraints	Δρ_min_ = −0.39 e Å^−^^3^

### Special details

**Table:**

Geometry. All esds (except the esd in the dihedral angle between two l.s. planes) are estimated using the full covariance matrix. The cell esds are taken into account individually in the estimation of esds in distances, angles and torsion angles; correlations between esds in cell parameters are only used when they are defined by crystal symmetry. An approximate (isotropic) treatment of cell esds is used for estimating esds involving l.s. planes.
Refinement. Refinement of F^2^ against ALL reflections. The weighted R-factor wR and goodness of fit S are based on F^2^, conventional R-factors R are based on F, with F set to zero for negative F^2^. The threshold expression of F^2^ > 2sigma(F^2^) is used only for calculating R-factors(gt) etc. and is not relevant to the choice of reflections for refinement. R-factors based on F^2^ are statistically about twice as large as those based on F, and R- factors based on ALL data will be even larger.

### Fractional atomic coordinates and isotropic or equivalent isotropic displacement parameters (Å^2^)

**Table:**

	*x*	*y*	*z*	*U*_iso_*/*U*_eq_	Occ. (<1)
C1	0.4404 (3)	0.8003 (3)	0.51805 (15)	0.0642 (9)	
H1	0.4892	0.8643	0.5167	0.077*	
C2	0.4434 (4)	0.7132 (4)	0.48146 (17)	0.0805 (12)	
H2	0.4927	0.7061	0.4501	0.097*	
C3	0.3080 (4)	0.6823 (4)	0.54427 (16)	0.0786 (12)	
H3	0.2460	0.6482	0.5640	0.094*	
C4	0.3724 (8)	0.5226 (7)	0.4718 (3)	0.0589 (17)	0.50
H4A	0.4425	0.5193	0.4480	0.071*	0.50
H4B	0.3766	0.4621	0.4998	0.071*	0.50
C5	0.2614 (8)	0.5143 (8)	0.4375 (4)	0.068 (2)	0.50
H5A	0.2580	0.5744	0.4094	0.082*	0.50
H5B	0.1916	0.5192	0.4615	0.082*	0.50
C4'	0.2942 (11)	0.5275 (8)	0.4825 (4)	0.088 (3)	0.50
H4'1	0.3111	0.4674	0.5095	0.105*	0.50
H4'2	0.2091	0.5410	0.4823	0.105*	0.50
C5'	0.3341 (11)	0.4935 (10)	0.4261 (4)	0.102 (4)	0.50
H5'1	0.4191	0.4801	0.4267	0.122*	0.50
H5'2	0.3183	0.5547	0.3995	0.122*	0.50
C6	0.2708 (5)	0.3860 (4)	0.40691 (19)	0.0887 (14)	
H6A	0.2973	0.3341	0.4363	0.106*	
H6B	0.1898	0.3641	0.3980	0.106*	
C7	0.3336 (4)	0.4423 (4)	0.3080 (2)	0.0847 (13)	
H7A	0.3863	0.4186	0.2778	0.102*	
H7B	0.3618	0.5156	0.3217	0.102*	
C8	0.3429 (4)	0.3571 (4)	0.3554 (2)	0.0870 (13)	
H8A	0.3178	0.2831	0.3415	0.104*	
H8B	0.4257	0.3506	0.3664	0.104*	
C9	0.2099 (4)	0.4585 (3)	0.28368 (16)	0.0683 (10)	
H9A	0.1563	0.4816	0.3137	0.082*	
H9B	0.2119	0.5193	0.2559	0.082*	
C10	0.2002 (3)	0.3110 (3)	0.20743 (14)	0.0615 (9)	
H10	0.2583	0.3444	0.1849	0.074*	
C11	0.0662 (3)	0.1975 (3)	0.23820 (14)	0.0592 (8)	
H11	0.0137	0.1364	0.2407	0.071*	
C12	0.0775 (3)	0.2814 (3)	0.27673 (14)	0.0615 (8)	
H12	0.0352	0.2887	0.3103	0.074*	
Zn1	0.31792 (3)	0.87724 (3)	0.625633 (16)	0.04733 (16)	
Cl1	0.43448 (8)	1.03207 (8)	0.61904 (4)	0.0671 (2)	
Cl2	0.12350 (7)	0.91670 (9)	0.63202 (3)	0.0642 (2)	
N1	0.3546 (2)	0.7805 (2)	0.55749 (10)	0.0532 (6)	
N2	0.3600 (5)	0.6368 (3)	0.49914 (15)	0.0942 (13)	
N3	0.1635 (3)	0.3541 (2)	0.25686 (11)	0.0557 (7)	
N4	0.1445 (2)	0.2161 (3)	0.19457 (10)	0.0533 (6)	

### Atomic displacement parameters (Å^2^)

**Table:**

	*U*^11^	*U*^22^	*U*^33^	*U*^12^	*U*^13^	*U*^23^
C1	0.0628 (19)	0.069 (2)	0.061 (2)	0.0015 (17)	0.0093 (16)	−0.0059 (16)
C2	0.099 (3)	0.080 (3)	0.062 (2)	0.024 (3)	0.011 (2)	−0.012 (2)
C3	0.121 (3)	0.058 (2)	0.057 (2)	−0.023 (2)	0.008 (2)	−0.0031 (17)
C4	0.063 (4)	0.060 (4)	0.053 (4)	0.013 (4)	0.005 (4)	−0.006 (3)
C5	0.065 (5)	0.091 (6)	0.050 (4)	0.025 (4)	0.000 (4)	0.005 (4)
C4'	0.107 (8)	0.073 (6)	0.084 (7)	−0.017 (6)	0.023 (6)	−0.022 (5)
C5'	0.089 (7)	0.101 (8)	0.116 (8)	−0.031 (6)	0.030 (6)	−0.072 (7)
C6	0.123 (4)	0.074 (3)	0.069 (3)	−0.021 (3)	−0.019 (2)	−0.0079 (19)
C7	0.078 (3)	0.090 (3)	0.087 (3)	−0.021 (2)	0.015 (2)	−0.008 (2)
C8	0.064 (2)	0.095 (3)	0.103 (3)	−0.007 (2)	−0.012 (2)	−0.003 (3)
C9	0.089 (3)	0.052 (2)	0.063 (2)	−0.0029 (19)	0.0126 (18)	−0.0050 (15)
C10	0.073 (2)	0.062 (2)	0.0498 (17)	−0.0024 (18)	0.0185 (15)	0.0018 (15)
C11	0.0557 (18)	0.062 (2)	0.0603 (19)	−0.0009 (16)	0.0095 (15)	0.0014 (15)
C12	0.0626 (19)	0.070 (2)	0.0517 (18)	0.0045 (19)	0.0181 (15)	−0.0010 (15)
Zn1	0.0504 (3)	0.0487 (3)	0.0429 (2)	0.00272 (17)	−0.00423 (16)	−0.00345 (15)
Cl1	0.0643 (5)	0.0595 (5)	0.0776 (5)	−0.0137 (4)	−0.0092 (4)	−0.0075 (4)
Cl2	0.0480 (4)	0.0808 (6)	0.0637 (5)	0.0066 (4)	−0.0064 (3)	−0.0068 (4)
N1	0.0620 (15)	0.0520 (16)	0.0457 (13)	−0.0003 (13)	0.0016 (11)	−0.0028 (11)
N2	0.176 (4)	0.0519 (18)	0.0544 (18)	0.001 (2)	0.001 (2)	−0.0125 (14)
N3	0.0665 (16)	0.0536 (16)	0.0470 (14)	0.0053 (13)	0.0101 (12)	−0.0004 (11)
N4	0.0555 (14)	0.0562 (17)	0.0481 (13)	0.0054 (13)	0.0080 (11)	0.0016 (11)

### Geometric parameters (Å, °)

**Table:**

C1—C2	1.338 (6)	C6—H6B	0.9700
C1—N1	1.362 (4)	C7—C8	1.505 (7)
C1—H1	0.9300	C7—C9	1.519 (6)
C2—N2	1.362 (6)	C7—H7A	0.9700
C2—H2	0.9300	C7—H7B	0.9700
C3—N1	1.302 (5)	C8—H8A	0.9700
C3—N2	1.329 (6)	C8—H8B	0.9700
C3—H3	0.9300	C9—N3	1.473 (5)
C4—C5	1.492 (12)	C9—H9A	0.9700
C4—N2	1.493 (8)	C9—H9B	0.9700
C4—H4A	0.9700	C10—N4	1.312 (5)
C4—H4B	0.9700	C10—N3	1.339 (4)
C5—C6	1.672 (10)	C10—H10	0.9300
C5—H5A	0.9700	C11—C12	1.346 (5)
C5—H5B	0.9700	C11—N4	1.374 (4)
C4'—C5'	1.464 (8)	C11—H11	0.9300
C4'—N2	1.531 (7)	C12—N3	1.372 (5)
C4'—H4'1	0.9700	C12—H12	0.9300
C4'—H4'2	0.9700	Zn1—N4^i^	2.008 (3)
C5'—C6	1.516 (10)	Zn1—N1	2.013 (3)
C5'—H5'1	0.9700	Zn1—Cl2	2.2415 (13)
C5'—H5'2	0.9700	Zn1—Cl1	2.2438 (12)
C6—C8	1.502 (7)	N4—Zn1^ii^	2.008 (3)
C6—H6A	0.9700		
			
C2—C1—N1	109.3 (4)	C8—C7—H7B	108.4
C2—C1—H1	125.3	C9—C7—H7B	108.4
N1—C1—H1	125.3	H7A—C7—H7B	107.5
C1—C2—N2	106.5 (4)	C6—C8—C7	114.6 (4)
C1—C2—H2	126.7	C6—C8—H8A	108.6
N2—C2—H2	126.7	C7—C8—H8A	108.6
N1—C3—N2	111.7 (4)	C6—C8—H8B	108.6
N1—C3—H3	124.1	C7—C8—H8B	108.6
N2—C3—H3	124.1	H8A—C8—H8B	107.6
C5—C4—N2	102.4 (6)	N3—C9—C7	112.7 (3)
C5—C4—H4A	111.3	N3—C9—H9A	109.1
N2—C4—H4A	111.3	C7—C9—H9A	109.1
C5—C4—H4B	111.3	N3—C9—H9B	109.1
N2—C4—H4B	111.3	C7—C9—H9B	109.1
H4A—C4—H4B	109.2	H9A—C9—H9B	107.8
C4—C5—C6	103.9 (6)	N4—C10—N3	112.0 (3)
C4—C5—H5A	111.0	N4—C10—H10	124.0
C6—C5—H5A	111.0	N3—C10—H10	124.0
C4—C5—H5B	111.0	C12—C11—N4	109.4 (3)
C6—C5—H5B	111.0	C12—C11—H11	125.3
H5A—C5—H5B	109.0	N4—C11—H11	125.3
C5'—C4'—N2	108.3 (7)	C11—C12—N3	106.7 (3)
C5'—C4'—H4'1	110.0	C11—C12—H12	126.6
N2—C4'—H4'1	110.0	N3—C12—H12	126.6
C5'—C4'—H4'2	110.0	N4^i^—Zn1—N1	107.47 (11)
N2—C4'—H4'2	110.0	N4^i^—Zn1—Cl2	105.25 (8)
H4'1—C4'—H4'2	108.4	N1—Zn1—Cl2	111.74 (8)
C4'—C5'—C6	110.8 (7)	N4^i^—Zn1—Cl1	111.93 (9)
C4'—C5'—H5'1	109.5	N1—Zn1—Cl1	106.23 (9)
C6—C5'—H5'1	109.5	Cl2—Zn1—Cl1	114.13 (5)
C4'—C5'—H5'2	109.5	C3—N1—C1	105.8 (3)
C6—C5'—H5'2	109.5	C3—N1—Zn1	127.4 (3)
H5'1—C5'—H5'2	108.1	C1—N1—Zn1	126.7 (2)
C8—C6—C5'	100.1 (5)	C3—N2—C2	106.7 (3)
C8—C6—C5	126.0 (5)	C3—N2—C4	138.5 (5)
C8—C6—H6A	105.8	C2—N2—C4	113.0 (5)
C5'—C6—H6A	99.3	C3—N2—C4'	109.2 (5)
C5—C6—H6A	105.8	C2—N2—C4'	143.5 (5)
C8—C6—H6B	105.8	C10—N3—C12	106.5 (3)
C5'—C6—H6B	136.6	C10—N3—C9	125.4 (3)
C5—C6—H6B	105.8	C12—N3—C9	128.1 (3)
H6A—C6—H6B	106.2	C10—N4—C11	105.5 (3)
C8—C7—C9	115.4 (4)	C10—N4—Zn1^ii^	123.3 (2)
C8—C7—H7A	108.4	C11—N4—Zn1^ii^	131.2 (3)
C9—C7—H7A	108.4		

Symmetry codes: (i) −*x*+1/2, −*y*+1, *z*+1/2; (ii) −*x*+1/2, −*y*+1, *z*−1/2.

### Hydrogen-bond geometry (Å, °)

**Table:**

*D*—H···*A*	*D*—H	H···*A*	*D*···*A*	*D*—H···*A*
C3—H3···Cl1^iii^	0.93	2.77	3.698 (5)	176

Symmetry codes: (iii) −*x*+1/2, *y*−1/2, *z*.
